# Serum Decorin, Biglycan, and Extracellular Matrix Component Expression in Preterm Birth

**DOI:** 10.1007/s43032-020-00251-1

**Published:** 2020-08-17

**Authors:** Jenna M. Mennella, Lori A. Underhill, Sophia Collis, Geralyn M. Lambert-Messerlian, Richard Tucker, Beatrice E. Lechner

**Affiliations:** 1 Warren Alpert Medical School at Brown University, Providence, RI USA; 2 Department of Pediatrics, Women and Infants Hospital, Providence, RI USA; 3 Brown University, Providence, RI USA; 4 Department of Pathology and Laboratory Medicine and Obstetrics and Gynecology, Women and Infants Hospital, Providence, RI USA

**Keywords:** Decorin, Biglycan, Preterm birth, PPROM, Biomarkers

## Abstract

Preterm birth is a leading cause of infant morbidity and mortality. Decorin and biglycan are proteoglycans that play key roles in maintaining the connective tissue matrix and tensile strength of human fetal membranes and have been previously linked to PPROM. Extracellular matrix proteins, such as matrix metalloproteinase 2 (MMP-2), matrix metalloproteinase 9 (MMP-9), TIMP metallopeptidase inhibitor 1 (TIMP-1), TIMP metallopeptidase inhibitor 2 (TIMP-2), and collagen VI (COL-6), have also been linked to PPROM and may have utility in a serum-based screening model for this condition. To define the natural course of serum decorin and biglycan expression throughout the duration of healthy pregnancy, to explore patterns of serum decorin and biglycan expression in serum of asymptomatic women who go on to develop spontaneous preterm labor, and to investigate the potential role for matrix metalloproteinases, their inhibitors, and collagen VI in a serum-based screening model to predict PPROM. Serum decorin level decreases less than 1% per week, and serum biglycan decreases by 2.9% per week over the duration of healthy pregnancy. Serum decorin and biglycan concentrations do not differ in spontaneous preterm labor cases compared with those in controls. Mean concentrations of MMP-2, MMP-9, TIMP-1, TIMP-2, and COL-6 do not differ in PPROM cases compared with those in controls. We have demonstrated that serum decorin and biglycan concentrations remain stable throughout the duration of normal pregnancy and are not early indicators of preterm labor, while common MMPs, TIMPs, and collagen VI are not early indicators of PPROM.

## Introduction

Preterm birth, defined as births occurring before 37 completed weeks of gestation, is a leading cause of infant morbidity and mortality [1, 2]. The burden of preterm birth is substantial. It affects approximately 11% of all livebirths worldwide [3] and is the most frequent cause of neonatal death [4]. It is also associated with both short-term and long-term neonatal morbidity [5, 6].

Various mechanisms lead to premature birth, and the majority of cases are spontaneous [7]. Women with spontaneous preterm birth typically present with preterm premature rupture of membranes (PPROM) or preterm labor with regular contractions and cervical dilation. PPROM is the spontaneous rupture of fetal membranes prior to labor before 37 weeks of gestation and accounts for about 30% of preterm births. Approximately 45% of preterm births follow spontaneous preterm labor. The remainder of preterm births are due to maternal or fetal indications, in which labor is induced or the infant delivered by cesarean section prior to labor onset [7].

PPROM and spontaneous preterm labor leading to preterm birth result from the dysregulation of complex pathways that are not entirely understood. They have been associated with a number of maternal factors, but their exact etiology remains unclear. Risk factors such as infection, inflammation, oxidative stress, nutritional deficiencies, multiple gestations, stress, and genetic predisposition have all been linked to both processes [8–11]. A prior preterm birth is also a major risk factor for subsequent preterm births [9]. Shortened cervical length may also be associated with increased risk for spontaneous preterm birth [12]. Behavioral and demographic risk factors such as poor socioeconomic status, smoking, and African ethnicity are associated with PPROM specifically [13–15]. Additionally, connective tissue disorders are associated with weakened fetal membranes and an increased incidence of PPROM [16].

Proteoglycans play key roles in maintaining the connective tissue matrix and tensile strength of many organs and participate in various cellular pathways [17, 18]. Decorin and biglycan are two highly homologous small leucine-rich proteoglycans (SLRPs) that are expressed in a variety of gestational tissues in humans and mice [19–21]. They are the most abundant proteoglycans expressed in human fetal membranes and play a role in maintaining the stability of the fetal membrane extracellular matrix [22, 23]. We have previously shown that decorin and biglycan transcriptionally compensate for each other in mouse fetal membranes by increasing decorin mRNA expression in biglycan-null fetal membranes and increasing biglycan mRNA expression in decorin null fetal membranes, but they lose their ability to do so in the presence of inflammation [24]. Mice deficient in decorin and biglycan have also been shown to deliver pups prematurely and display fetal membrane structural abnormalities [22, 25].

Due to the complex pathways that lead to spontaneous preterm labor and PPROM, there are currently no treatments for these disorders and no exact methods available to predict their occurrence. Insulin-like growth factor-binding protein 4 (IGFBP-4) and serum protein sex hormone-binding globulin (SHBG) are two serum proteins that were recently identified as predictors of spontaneous preterm birth [26], but they do not distinguish between the various causes of spontaneous preterm birth.

In early pregnancy serum samples from asymptomatic women who later developed PPROM, we showed elevated biglycan serum concentrations in conjunction with decreased decorin and SHBG serum concentrations [27]. These three biomarkers were analyzed separately and in combination to determine their predictive capacity, with the combined 3-variable model showing an area under the curve (AUC) of 0.774, making it a satisfactory model to predict PPROM [27]. Biomarkers such as these allow for early clinical intervention in asymptomatic women at high risk for preterm birth. However, multiple questions regarding the use of these biomarkers remain unaddressed. We do not know the serum expression profile of these proteins throughout the course of healthy pregnancy, as well as their patterns in spontaneous preterm labor without PPROM. It is unclear if the patterns exhibited by decorin and biglycan in serum of women who later go on to develop PPROM are specific to the pathophysiology of PPROM or whether they are similar to the patterns exhibited in women who go on to develop spontaneous preterm labor.

Additionally, other markers that have been linked to PPROM may play a role in the refinement of this biomarker model for PPROM. For example, other extracellular matrix proteins that have been linked to PPROM are matrix metalloproteinase-2 (MMP-2) and matrix metalloproteinase-9 (MMP-9), two proteolytic enzymes that help regulate matrix remodeling and degradation [28, 29]. Elevated MMP-2 and MMP-9 amniotic fluid concentrations have been found to be associated with PPROM [30, 31], and amniotic fluid levels of the endogenous inhibitors of matrix metalloproteinases (TIMPs) are also abnormal in PPROM, with increased TIMP-1 levels and decreased TIMP-2 levels compared with rupture of membranes at term [31]. Collagen VI is a structural protein that has been shown to interact closely with decorin and biglycan in the extracellular matrix of various tissues. Concentrations of these markers in the serum of pregnant women may contribute to a serum-based screening model for PPROM but have yet to be examined.

Thus, the aims of this study were threefold: (1) to define the natural course of serum decorin and biglycan throughout the duration of healthy pregnancy, (2) to explore patterns of serum decorin and biglycan expression in serum of asymptomatic women who go on to develop spontaneous preterm labor, and (3) to investigate the potential role for matrix metalloproteinases, their inhibitors, and collagen VI in a serum-based screening model to predict PPROM.

## Methods

Serum samples were collected from patients at Women & Infants Hospital in Providence, Rhode Island. Women & Infants Hospital Institutional Review Board approval was obtained.

### Uncomplicated Pregnancy Serum Samples

Residual serum was collected from the Women & Infants Hospital laboratory. Analyzed samples came from women who presented for prenatal diagnostic testing at various points in pregnancy, those who presented to the emergency department for issues unrelated to pregnancy, and those who were admitted to the hospital in the peripartum period. The electronic medical record was used to identify gestational age at the time of serum collection. Samples were excluded if the pregnancy was not low risk. Specifically, samples were excluded if women reported any pregnancy complications, such as pain or contractions, leakage of amniotic fluid, or concern for chorioamnionitis. Pregnancies with multiple gestations or congenital anomalies were also excluded. Additionally, three samples from women who were post-partum and three samples from non-pregnant women were also identified using the electronic medical record and included in the analysis. Samples were screened between January 2018 and April 2018. These serum samples were stored at 4 °C and were screened 6 days after collection, at which point samples are routinely discarded by the lab. No significant difference was found between serum samples analyzed on day 0 vs. day 6 after storage in 4 °C refrigerator for both decorin and biglycan (data not shown).

### Preterm Labor and PPROM Serum Samples with Matched Controls

For the preterm labor and PPROM samples, residual serum was collected from women who presented for second trimester screening. For all cases, chart review was performed to identify the first 20 patients with preterm labor and the first 18 patients with PPROM going backward temporally from 2016. Preterm labor samples were from November 2014 to October 2016. PPROM samples were from February 2014 to February 2017. Preterm labor was defined as contractions or pelvic pain associated with cervical change. Cases in which leakage of fluid or infection coincided with preterm labor were excluded from the preterm labor group. PPROM was defined as presentation with rupture of fetal membranes and/or leakage of amniotic fluid as the first concerning sign or symptom in an otherwise uncomplicated pregnancy. Cases in which contractions, pain, or infection were documented as symptoms prior to or coinciding with leakage of fluid were excluded from the PPROM group. Five matched controls were identified for each case. The control samples were matched for gestational age at sample collection (within the same completed week, 15–20 weeks of gestation), maternal race (African American or non-African American), and duration of freezer storage (± 6 months) based on previous preanalytical studies indicating differences among these factors [27]. None of the patients was smokers. From collection to analysis, serum was stored at − 20 °C.

### Assays

Serum biglycan and decorin were measured using commercially available enzyme-linked immunoabsorbent sandwich assay (ELISA) kits (Human Biglycan ELISA kit, Clone-Cloud Corp, Houston, Texas; and Human Decorin ELISA kit, Sigma-Aldrich, St. Louis, Missouri). The assays were performed according to the manufacturer’s instructions. Standard validation of the biglycan and decorin immunoassays was performed to assess for linearity and effect of storage duration. Serum MMP2, MMP9, TIMP1, TIMP2, and COL6 were measured using commercially available ELISA kits, according to the manufacturer’s instructions (Human MMP2 and COL6 ELISA kits, antibodies-online; Human MMP9, TIMP1, and TIMP2 ELISA kits, Sigma-Aldrich, St. Louis, Missouri). Inter-assay coefficients of variation (COV) and assay sensitivities (AS) for ELISAs are as follows: biglycan COV < 12%, AS 0.127 ng/ml; decorin COV < 12%, AS 1.5 pg/ml; MMP2 COV < 12%, AS 12.1 pg/ml; MMP9 COV < 12%, AS 10 pg/ml; TIMP1 COV < 12%, AS 40 pg/ml; TIMP2 COV < 12%, AS 2 pg/ml; and COL6 < 10%, AS 4.69 ng/ml. The ELISA process for all assays entailed the following steps: (1) a monoclonal capture antibody is precoated onto 96-well plates; (2) the biological sample and standard are then added to the well and incubated; (3) biotin-labeled detection antibody is added to the well, followed by incubation with horseradish peroxidase (HRP) streptavidin; (4) unbound conjugates are washed away; (5) colorimetric analysis is performed on a Bio-Rad iMark Microplate Reader (Bio-Rad, Hercules, California) at 450 nm. Testing of uncomplicated pregnancy samples was performed without operator knowledge of gestational age or pregnant/non-pregnant status. Testing of PPROM and preterm labor samples was performed without operator knowledge of the outcome group.

### Statistical Analysis

For healthy pregnancy samples, data for biglycan and decorin were fit by negative binomial regression with week as the independent variable. Changes in levels by week were expressed as rate ratios indicating percent change. For PPROM and preterm labor, comparisons of biglycan and decorin on the matched case/control sample were made with negative binomial models with generalized estimating equations (GEE) to adjust for matching. The additional PPROM markers, which approximated a normal distribution, were analyzed using linear models, with GEE to adjust for matching.

## Results

### Uncomplicated Pregnancy Serum Samples

**Fig. 1:**
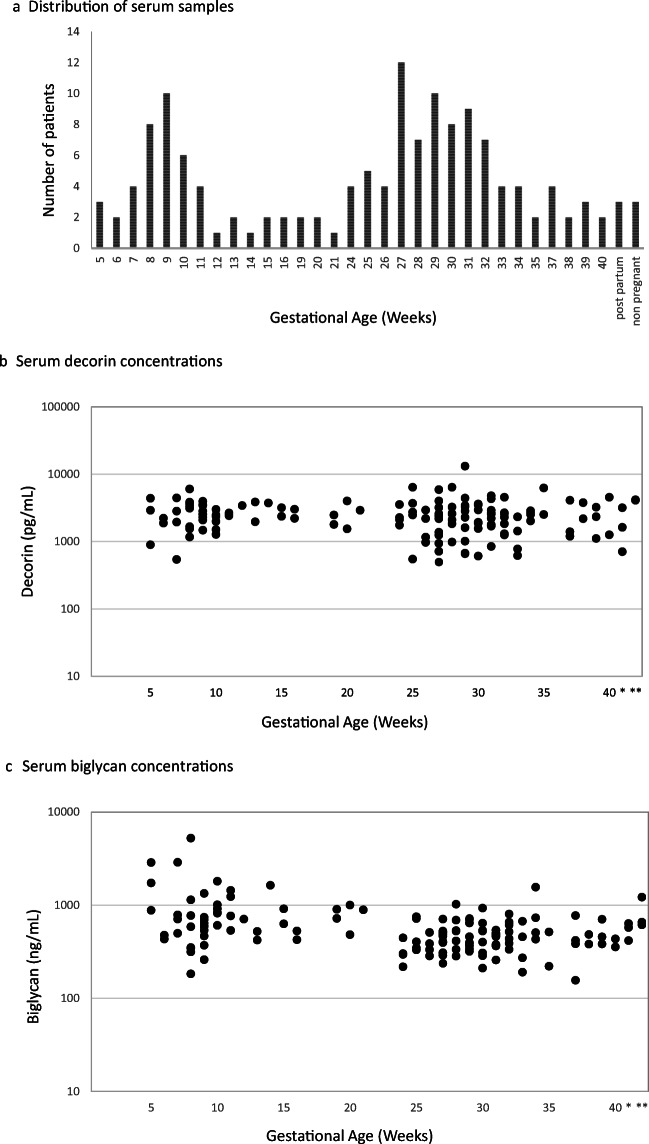
**a** Distribution of serum samples. **b** Serum decorin concentrations. **c** Serum biglycan concentrations. 1A. Distribution of analyzed serum samples by gestational age in weeks (*n* = 143). 3 samples were from women who were post-partum and 3 from non-pregnant women. 1B. Serum decorin concentrations by gestational age (log scale). Decorin concentrations are stable during the course of gestation, decreasing by less than 1% per week (*p* = 0.608). Asterisk and double asterisk on the x-axis represent samples from post-partum and non-pregnant women, respectively. 1C. Serum biglycan concentrations by gestational age (log scale). Biglycan levels decrease by 2.9% per week (*p* = 0.0001). Asterisk and double asterisk on the x-axis represent samples from post-partum and non-pregnant women, respectively

The analyzed samples were from patients between 5 and 40 weeks of gestation, plus three additional post-partum and non-pregnant patients. A total of 143 serum samples were analyzed, with distribution of gestational ages shown in Fig. 1a. Figure 1b and c demonstrate patterns of serum decorin and biglycan concentrations over the duration of uncomplicated pregnancy. Serum decorin level decreased by less than 1% per week (*p* = 0.608) and serum biglycan decreased by 2.9% per week (*p* = 0.0001).

### Preterm Labor Marker Study

**Fig. 2:**
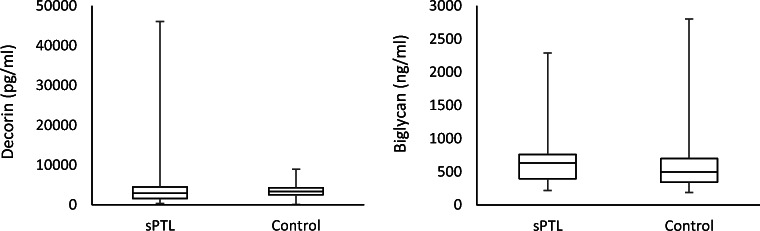
Serum decorin and biglycan levels in spontaneous preterm labor (sPTL). Early pregnancy serum decorin and biglycan levels in spontaneous preterm labor (sPTL) and controls. Mean serum decorin and biglycan levels do not differ in sPTL compared with those in controls (*p* = 0.38 and *p* = 0.34 for decorin and biglycan, respectively)

Given that we previously demonstrated a significant difference in serum decorin and biglycan concentrations in asymptomatic women who went on to develop PPROM compared with those in matched controls who delivered at term, as a next step, we tested whether this pattern is similar in spontaneous preterm labor without rupture of membranes in order to ascertain whether the reported results were related to preterm birth in general or PPROM specifically. Gestational age at diagnosis of preterm labor ranged between 20 and 32 weeks of gestation. Gestational age at birth also ranged from 20 to 32 weeks of gestation. Of the 20 cases of preterm labor, 6 infants were born at a previable gestational age, while 14 were viable with subsequent admission to the NICU. Two infants died in the NICU secondary to morbidities associated with prematurity, and 12 infants survived to discharge. The median maternal age in years was 28 (range: 18 to 43) for cases with preterm labor and 29 (range: 17–33) for controls. Cases and controls were matched for gestational age. Median gestational age at the time of serum sampling was 17.07 weeks for preterm labor cases and 17.14 weeks for controls. Mean decorin and biglycan levels do not differ in the spontaneous preterm labor cases compared with those in controls (Fig. 2; *p* = 0.38 and *p* = 0.34 for decorin and biglycan, respectively).

### PPROM Additional Marker Study

**Fig. 3:**
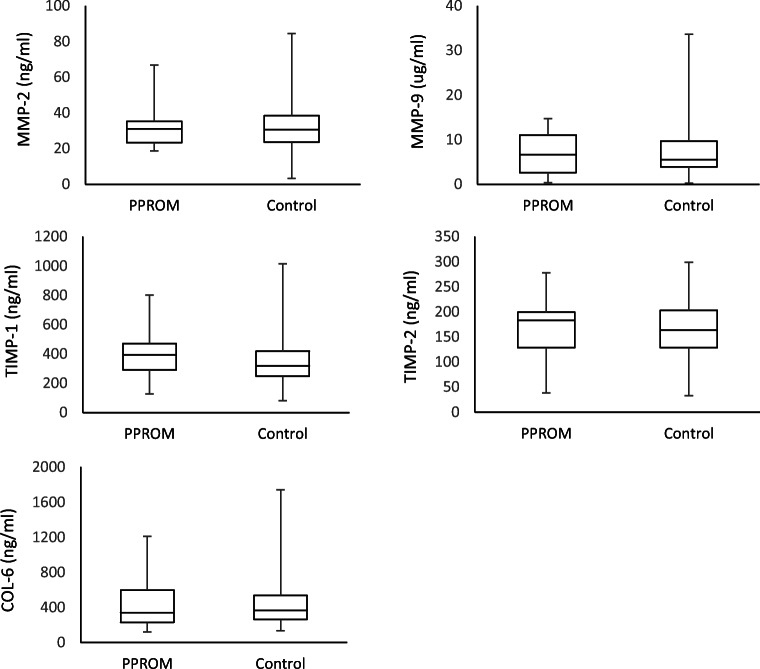
Early pregnancy serum extracellular matrix component levels in preterm premature rupture of membranes (PPROM). Early pregnancy serum extracellular matrix component levels in preterm premature rupture of membranes (PPROM) and controls. Mean concentrations of matrix metalloproteinase 2 (MMP-2), matrix metalloproteinase 9 (MMP-9), TIMP metallopeptidase inhibitor 1 (TIMP-1), TIMP metallopeptidase inhibitor 2 (TIMP-2), and collagen VI (COL-6) were unchanged in PPROM compared with those in controls (*p* = 0.91, *p* = 0.73, *p* = 0.25, *p* = 0.42, and *p* = 0.85 respectively)

Next, we examined serum levels of proteins known to play a role in the pathogenesis of PPROM to assess whether they demonstrated abnormal levels in the serum of women who went on to develop PPROM compared with those in matched controls. Eighteen cases of PPROM were analyzed. Gestational age at diagnosis of PPROM ranged from 17 to 27 weeks. Gestational age at birth ranged from 17 to 34 weeks of gestation. Of the 18 cases of PPROM, 8 infants were born at a previable gestational age and 10 were viable with subsequent admission to the NICU. All 10 infants survived to discharge. The median maternal age in years was 28.55 (range: 18–40) for cases with PPROM and 28.77 (range: 17–42) for controls. Cases and controls were matched for gestational age. The median gestational age at the time of the blood serum sampling was 16.5 weeks for cases with PPROM and 16.4 weeks for controls. Mean serum levels of the five proteins examined are presented in Fig. 3. Mean concentrations of matrix metalloproteinase 2 (MMP-2), matrix metalloproteinase 9 (MMP-9), TIMP metallopeptidase inhibitor 1 (TIMP-1), TIMP metallopeptidase inhibitor 2 (TIMP-2), and collagen VI (COL-6) were not significantly different in cases with PPROM compared with those in controls (*p* = 0.91, *p* = 0.73, *p* = 0.25, *p* = 0.42, and *p* = 0.85 respectively).

## Discussion

In this study, we have demonstrated the natural course of serum decorin and biglycan expression throughout the duration of pregnancy. We have also shown that serum decorin and biglycan do not exhibit abnormal expression patterns in early pregnancy prior to the occurrence of spontaneous preterm labor as they do in PPROM, where serum decorin concentrations are decreased and biglycan increased [27]. Lastly, we have shown that the expression of extracellular matrix proteins known to be associated with PPROM as well as with decorin and biglycan metabolism (MMP-2, MMP-9, TIMP-1, TIMP-2, collagen VI) does not display abnormal patterns in the early pregnancy serum of women who go on to develop PPROM. These findings strengthen our previously proposed model of decorin and biglycan as early serum markers of PPROM [27].

Given decorin and biglycan’s promising role as biomarkers of PPROM risk, it was necessary to elucidate the ontogeny of both proteoglycans throughout normal pregnancy. No previous studies have examined their baseline serum concentrations during gestation. Our results indicate that serum decorin and biglycan concentrations remain stable throughout the duration of pregnancy. This finding is consistent with the context of the placental role in mediating various pathways to adverse pregnancy outcomes. The placenta may secrete certain factors that are either passive indicators of its disrupted function or active molecules that regulate maternal physiology [32]. In abnormal pregnancies, these factors may be detectable in the maternal circulation and can potentially lend to the early detection of pathology. Thus, stable concentrations of serum decorin and biglycan throughout normal pregnancy further support their role as predictors of PPROM.

PPROM and preterm labor are discrete pathologic processes that can lead to spontaneous preterm birth. There is much overlap in the etiologic factors that predispose to these conditions; however, their pathophysiologic processes are distinct, and it remains unclear why some women are susceptible to PPROM and others to preterm labor [33]. Both processes have been linked to infection and inflammation [34–37], as well as to connective tissue abnormalities [16, 38, 39], but the exact pathway to their occurrence is variable and not completely understood. In addition, these processes exhibit much overlap in their clinical presentations. Thus, to clearly differentiate between a predictor of risk for PPROM versus for preterm labor versus for the end result of preterm birth, it was important to investigate early pregnancy serum decorin and biglycan levels prior to the onset of preterm labor later in pregnancy, in order to evaluate whether expression patterns are similar to those seen prior to PPROM.

Atalay et al. investigated serum decorin concentrations in women at the time of presentation with preterm labor between 24- and 32-week gestation and reported that median serum decorin levels were significantly lower in cases of preterm labor compared with those in controls [40]. In our study, we did not find significant differences in early pregnancy serum concentrations for either decorin or biglycan, suggesting that they do not have a role in predicting preterm labor before labor has commenced.

Serum biglycan levels have been studied in relation to other diseases, such as hepatitis B and endometrial cancer [41, 42], but not in relation to preterm labor. We previously demonstrated that serum biglycan concentrations in early pregnancy are increased prior to presentation with PPROM when compared with those in controls that went on to deliver at term [27]. Conversely, we found no significant difference in early serum concentrations in cases that went on to develop preterm labor.

Various markers of preterm labor have been discussed in the literature. Fetal fibronectin is an extracellular matrix glycoprotein that is detected in cervical and vaginal secretions during pregnancy [43]. Elevated levels of fetal fibronectin have been associated with increased risk for spontaneous preterm birth, specifically in nulliparous women, at the time of presentation with symptoms of preterm labor [44–46]. Interleukin-10 (IL-10) is a key cytokine in pregnancy that has also been implicated in preterm birth. Decreased concentrations of IL-10 in the placenta and increased amniotic fluid concentrations of IL-10 have both been observed in preterm labor [47, 48]. As suggested by the authors, a primary deficiency of placental IL-10 triggers the intrauterine inflammatory environment and increased production of pro-inflammatory mediators, causing a feedback upregulation of molecules such as IL-10 in amniotic fluids in response [48]. Additionally, monocyte chemotactic protein-1 (MCP-1) is a pro-inflammatory cytokine in amniotic fluid that is significantly associated with preterm birth in asymptomatic women with short cervix [49].

Because little is known about the mechanisms that lead to PPROM, biomarkers with predictive capacity are important to indicate susceptibility to this event. Unfortunately, there is a paucity of literature on such markers. A study by Sisti et al. found serum concentrations of insulin-like growth factor-binding protein 1 (IGFBP-1) in twin pregnancies prior to 20-week gestation to be significantly higher in cases prior to the development of PPROM, suggesting its potential role in early prediction of PPROM in this specific clinical scenario [50]. Serum ferritin levels are also significantly elevated in cases of PPROM at the time of presentation when compared with those in controls [51] compared with those in cases of preterm labor, in which there was no significant difference.

MMP-2 and MMP-9 are endopeptidases which play a role in the degradation of the extracellular matrix and are important factors in the remodeling process of human fetal membranes that occurs throughout the duration of pregnancy [29, 52]. Their activity is partially regulated by tissue inhibitors of metalloproteinases (TIMPs), and dysregulation of these enzymes has been previously linked to PPROM [31]. In the amniotic fluid of women with PPROM, levels of MMP-2, MMP-9, and TIMP-1 are significantly increased compared with those in term controls, and levels of TIMP-2 are significantly decreased [31]. Additionally, serum concentrations of MMP-2 and MMP-9 immediately after delivery in cases of PPROM have been noted to be significantly higher compared with those in controls [53]. These findings led us to investigate their concentrations in early pregnancy serum of women later diagnosed with PPROM, to evaluate their potential use in our predictive model. We did not find significant differences in serum concentrations of any of these markers when comparing PPROM cases and controls, suggesting that their dysregulation occurs closer to the time of membrane rupture and thus cannot be used to predict PPROM in asymptomatic women in early pregnancy.

Collagens are a major component of the fetal membrane extracellular matrix that undergo constant turnover during pregnancy as a target of MMPs [54]. Collagen VI is a ubiquitous extracellular matrix component that is known to interact tightly with decorin and biglycan [55]. In our analyses, we did not find significant differences in early pregnancy serum concentrations of collagen VI in future PPROM cases when compared with those in controls that delivered at term.

A caveat to the findings described in this study is the fact that while residual serum used for the healthy pregnancy analysis was collected from women whose pregnancies were uncomplicated up until the point of collection, subjects were not followed prospectively throughout full gestation. However, given that our samples were randomly collected and thus represented the general population in the hospital’s catchment area, and approximately 10% of the population delivers prematurely, plus many of the samples were collected late in pregnancy, it is reasonable to assume that the majority of our samples correlated to healthy full-term pregnancies. In addition, the retrospective analysis of medical records constitutes a challenge in clearly distinguishing between PPROM and preterm labor samples given the clinical overlap associated with these conditions. Lastly, it would be informative to examine the patterns of these biomarkers in the fetal membranes, to determine if the patterns we have observed in the serum are a reflection of the pathological occurrences in the placental tissues.

Future directions include a prospective study exploring serum decorin and biglycan concentrations in healthy pregnancies, pregnancies affected by PPROM, and pregnancies affected by preterm labor. A prospective study design will allow us to follow subjects throughout gestation and investigate the clinical circumstances surrounding pregnancy complications if and when they occur to enable the clear differentiation between cases of preterm membrane rupture without labor and cases of preterm labor without rupture of membranes. Confirmation of the significant variations in serum decorin and biglycan concentrations in cases of PPROM would strengthen their potential use as early pregnancy predictive markers of PPROM. Thus, future steps include the measurement of biglycan and decorin in fetal membranes at the time of birth.

In conclusion, we have demonstrated that serum decorin and biglycan concentrations remain stable throughout the duration of normal pregnancy and are not early indicators of preterm labor, while common MMPs, TIMPs, and collagen VI are not early indicators of PPROM. These findings strengthen the potential of early pregnancy serum decorin and biglycan levels as a predictive model for PPROM.
